# Complete factorial design to adjust pH and sugar concentrations in the inoculum phase of *Ralstonia solanacearum* to optimize P(3HB) production

**DOI:** 10.1371/journal.pone.0180563

**Published:** 2017-07-12

**Authors:** Karine Laste Macagnan, Mariane Igansi Alves, Amanda Ávila Rodrigues, Lígia Furlan, Rosane da Silva Rodrigues, Patrícia Diaz de Oliveira, Claire Tondo Vendruscolo, Angelita da Silveira Moreira

**Affiliations:** 1 Centro de Desenvolvimento Tecnológico, unidade de Biotecnologia, Universidade Federal de Pelotas, Pelotas, Rio Grande do Sul, Brasil; 2 Departamento de Ciência e Tecnologia Agroindustrial, Universidade Federal de Pelotas, Pelotas, Rio Grande do Sul, Brasil; 3 Centro de Ciências Químicas, Farmacêuticas e de Alimentos, Universidade Federal de Pelotas, Pelotas, Rio Grande do Sul, Brasil; Universite Paris-Sud, FRANCE

## Abstract

Poly(3-hydroxybutyrate) (P(3HB)) is a biodegradable plastic biopolymer that accumulates as lipophilic inclusions in the cytoplasm of some microorganisms. The biotechnological process by which P(3HB) is synthesized occurs in two phases. The first phase involves cell growth in a complex culture medium, while the second phase involves polymer accumulation in the presence of excess carbon sources. As such, the efficiency of the second phase depends on the first phase. The aim of this study was to evaluate culture media with different concentrations of sucrose and glucose and different pH values in the inoculum phase of *Ralstonia solanacearum* RS with the intention of identifying methods by which the biomass yield could be increased, subsequently enhancing the yield of P(3HB). The culture medium was formulated according to the experimental planning type of central composite rotational design 2^2^. The independent variables were pH and sugar concentration (sucrose and glucose), and the dependent variables were OD_600nm_, dry cell weight (DCW), and P(3HB) yield. The highest cell growth, estimated by the OD_600nm_ (20.6) and DCW (5.35) values, was obtained when sucrose was used in the culture medium at a concentration above 35 g.L^-1^ in combination with an acidic pH. High polymer (45%) accumulation was also achieved under these conditions. Using glucose, the best results for OD_600nm_ (12.5) and DCW (2.74) were also obtained at acidic pH but with a sugar concentration at the minimum values evaluated. Due to the significant accumulation of polymer in the cells that were still in the growth phase, the accumulating microorganism P(3HB) *Ralstonia solanacearum* RS can be classified as having type II metabolism in relation to the polymer accumulation phase, which is different from other *Ralstonia* spp. studied until this time.

## Introduction

Conventional plastics derived from petroleum have been in use for decades due to their strength, durability and low production costs [1]. While plastics are a major benefit to society, their continued use is questionable because they can have a serious impact on human health and the environment [2–3]. The growing scientific interest in the environmental impact of humanity’s increasing consumption of plastics suggests that there is a distinct requirement to research and develop environmentally friendly substitutes. A primary line of research into alternative materials that has attracted significant interest is the production of biodegradable biopolymers derived from microorganisms as renewable sources that offer the thermal and mechanical characteristics required for industrialization [4].

Bioprocesses can be used to obtain biodegradable polymers, such as the polyhydroxyalkanoate (PHA) family. PHAs accumulate in the cytoplasm of bacteria as inclusions of water-insoluble polyesters and as intracellular carbon and energy storage compounds [5]. The main characteristic of these bioplastics is that the enzymatic action of microorganisms completely degrades the plastics in a short time under suitable environmental conditions; in addition, they are thermoplastic and biocompatible with the human body [6]. These macromolecules have many applications, from food packaging to agricultural and medical uses; e.g., in drug delivery systems, implants, non-woven patches, and tissue scaffolding [7–9].

As P(3HB) has physical properties that are similar to those of polypropylene, including the melting point, crystallinity and glass transition temperature, it is the most studied and the most frequently used biopolymer for the production of bioplastics [10]. P(3HB) is produced via two phases. The first phase, named the “inoculum phase” or “cell growth”, occurs in a complex culture medium, usually nutrient broth (NB) [11–13], nutrient rich (NR) [14], basal culture medium (BCM) [15–16] or yeast malt (YM) [17] compounds of peptone and yeast, malt and meat extracts and complemented with a low C/N ratio. The second phase, named “polymer accumulation or production” occurs in mineral salt medium (MSM) [11–16,18–19] under the limitation of essential nutrients N, P, O, or Mg and in the presence of excess carbon sources associated with a high C/N ratio [11].

During the inoculum phase of P(3HB)-producing bacteria, it is important to maximize cell growth and achieve a high cell density. Inoculation of the cells grown in a specific new culture medium induces maximal accumulation of biopolymers with a minimal residual weight of the cell. Thus, polymer synthesis during the inoculum phase is not a prerequisite [20].

While over 300 microorganisms can synthesize PHAs, the production of PHAs is usually limited to *Ralstonia* spp., *Cupriavidus necator*, *Pseudomonas* spp. and recombinant *Escherichia coli* [9,19,21–22]. *Ralstonia* spp. tend to be more amenable to production on an industrial scale, demonstrating high yields and production rates [23] and accumulating approximately 80% of their dry weight as a polymer [24].

The strain used in the present study, *R*. *solanacearum* RS, is a phytopathogenic bacterium P(3HB) producer that was isolated from a cactus in Rio Grande do Sul (RS) and was characterized by 16S rRNA sequencing [25–26]. Albeit the *Ralstonia* genus is considered to be a good producer of exopolysaccharides, in our researcher group, the main interest about *R*. *solanacearum* RS has been focused on the novelty of studying this specie for the production of intracellular biopolymers, such as PHBs. The aim of this study was to evaluate the influence the carbon source concentration (sucrose and glucose) and initial pH of the culture medium had on cell growth during the inoculum phase.

## Materials and methods

### Microorganism

The *R*. *solanacearum* RS strain was supplied by the Bacteriology Laboratory, Eliseu Maciel Faculty of Agronomy, Federal University of Pelotas, RS, Brazil. The bacteria were lyophilized and stored at -80°C or sub-cultured monthly on nutritive yeast agar (NYA) [27] composed of (in g.L^-1^) peptone (Kasvi^®^), 5.0; glucose (Synth^®^), 5.0; yeast extract (Kasvi^®^), 1.0; meat extract (Himedia^®^), 3.0; and agar (Kasvi^®^), 15.0. They were then stored at 4°C.

### Culture media and operating conditions

The different culture media for the inoculum phase were obtained by combining the independent variables according to the experimental plan. The processes were conducted in an orbital shaker incubator with yeast malt [28] culture medium composed of (in g.L^-1^) yeast extract (Kasvi^®^), 2.7; malt extract (Kasvi^®^), 2.7 and peptone (Kasvi^®^), 4.5, modified based on the sugar type, concentration and pH.

The processes were conducted in 250 mL Erlenmeyer flasks with **a** total volume of 100 mL. The inoculum, with initial DO_600nm_ 0.5, was produced from pre-inoculums formed by the suspension of fresh cells obtained from multiplicative cultures on NYA [27] solid medium plates for 72 h at 32°C. The incubations occurred at 32°C with shaking at 250 rpm for 24 h.

Two experimental designs were used for each type of sugar (glucose (Synth^®^) and sucrose (Synth^®^) to establish the working ranges and the best experimental conditions. Initially, a complete factorial design 2^2^ was used with three levels (-1, 0, +1) and three central points, and the independent variables were pH (5 to 8) and sugar concentration (0 to 70 g.L^-1^), totalling seven treatments. The results of the first statistical treatment were analysed, and the best range was established.

Next, a second experimental design was proposed central composite rotational design (CCRD) 2^2^, with four experiments at the axial points and three at the central point, totalling 11 treatments for each type of sugar (glucose and sucrose). The glucose solution ranged in pH (5.5 to 9.5) and sugar concentration (10 to 50 g.L^-1^), and the sucrose solution ranged in pH (5.8 to 7.2) and sugar concentration (15 to 45 g.L^-1^).

The culture medium pH adjustment was made with 2 M solutions of HCl or NaOH. The culture media were autoclaved at 121°C for 15 min, and the sugar solutions were sterilized separately and added to the culture medium later.

### Determination of total residual sugars

Residual glucose and sucrose from the cultures were determined by the dinitrosalicylic acid (DNS) method for reducing sugars [29]. Samples from cultures with sucrose, a non-reducing sugar, were hydrolysed with 2 M HCl and then neutralized with 2 M NaOH. The supernatants were diluted 1:40 or 1:50 (v.v^-1^) as needed. The procedure was done in a test tube with 1 mL of sample added with 1 mL of the DNS reagent. The samples were stirred and heated at 100°C for 5 min; they were then transferred to cooling for 5 min, and 16 mL of sodium and potassium double tartrate solution were added. The readings were carried out in a spectrophotometer at 540 nm. The standard curve was constructed with glucose ranging from 0 to 1.0 g.L^-1^.

### Determination of cell growth and accumulation of P(3HB)

Cell growth was evaluated by optical density (OD_600nm_), which was measured by spectrophotometry at 600 nm. The dry cell weight concentration (DCW) was determined by gravimetry. Following the accumulation step for P(3HB) production, the bacteria were harvested by centrifugation (10,000 ×g for 15 min), the cell concentrate was resuspended in 0.89% saline solution, and the biomass was recovered (10,000 ×g for 10 min). The DCW was obtained by drying at 56°C.

The accumulation of P(3HB) was determined by chemical extraction using chloroform and the DCW in the ratio of 40:1 v.m^-1^ [30]. After their recovery, the films were weighed for the yield calculation, which was expressed as a percentage. Eq 1 was used to determine the yield.

%Y = (P1 ÷ P2) × 100 (1)

where P1 is the total weight of the recovered biofilm and P2 is the DCW.

All averages resulted from triplicate measurements. Comparisons were statistically analysed with an ANOVA test using the Statistica 7.0 program. A value of p <0.05 was considered to be significant.

## Results and discussion

**Fig 1:**
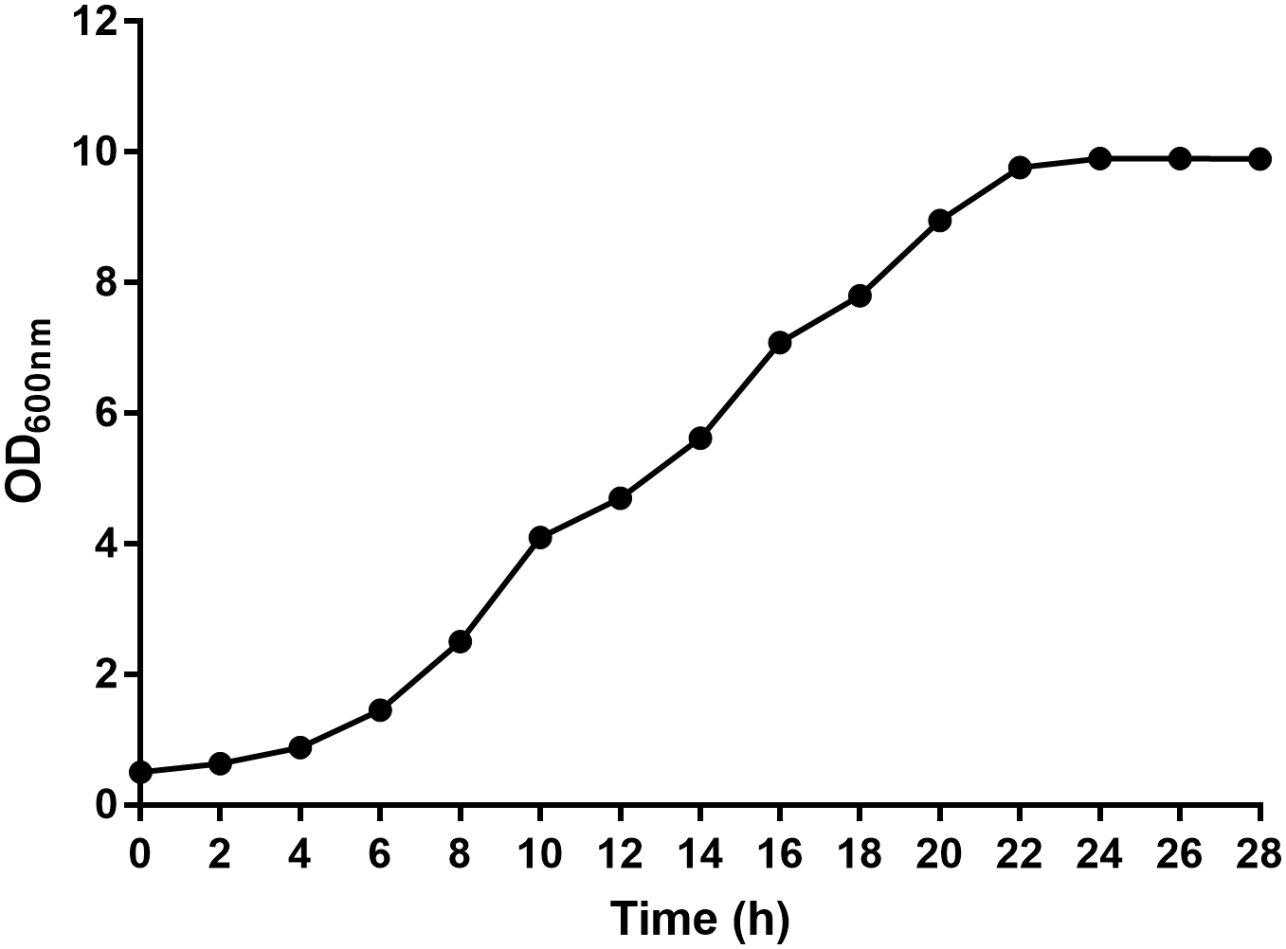
Growth curve of the *Ralstonia solanacearum* RS strain.

The growth curve of the *Ralstonia solanacearum* RS strain was determined to explain its development in relation to the fermentation time (Fig 1). It was observed that the stationary phase was between 22 h and 28 h of cultivation Therefore, we selected the 24 h time to discuss the OD and DCW results and perform P(3HB) recovery and sugar consumption.

### Complete factorial design 2^2^

The matrix of the complete factorial design 2^2^ is presented in Table 1 and was used to evaluate the effect of the sugar concentration (glucose or sucrose) and the pH of the culture medium on the cellular growth of *Ralstonia solanacearum* RS, expressed through OD_600nm_.

**Table 1:** Matrix of the complete factorial design 2^2^. Coded and real levels as well as response variables obtained for *R*. *solanacearum* RS inocula incubated at 32°C and 250 rpm for 24 h in YM culture medium containing different concentrations (g.L^-1^) of glucose or sucrose.

Treatments	Variable		OD_600nm_	
	pH	[Sugar]	Glucose	Sucrose
1	-1 (5)	-1 (0)	7.3	4.2
2	+1 (8)	-1 (0)	6.8	6.5
3	-1 (5)	+1 (70)	5.0	6.0
4	+1 (8)	+1 (70)	8.7	7.1
5	0 (6.5)	0 (35)	7.6	21.2
6	0 (6.5)	0 (35)	8.1	21.9
7	0 (6.5)	0 (35)	8.2	18.9

OD_600nm_, optical density.

Glucose was used to verify whether the pH and combination of the variables had a positive effect on the biomass, as the sugar concentration had no significant isolated effect. The highest cell growth obtained for glucose (OD_600nm_ 8.7) was achieved via a combination of +1/+1 of pH 8 and 70 g.L^-1^ sugar. The highest cell growth obtained for sucrose (OD_600nm_ 20.7) was achieved using the combination of pH 6.5 and a sugar concentration of 35 g.L^-1^. When sucrose was used, the independent variables had no effect in the studied ranges; however, the highest biomass concentrations were obtained when this sugar was used. This finding is very relevant given that sucrose is available at a lower price than glucose in Brazil.

From the results obtained in this complete factorial planning, a central composite rotational design (CCRD) was developed to obtain response surfaces that indicated the best combinations of pH and sugar concentration.

### Central composite rotational design 2^2^

Table 2 presents the results generated by the application of the central composite rotational design (CCRD 2^2^) statistical design. The design was based on the response surface methodology (RSM), which was usped to evaluate the effect of sucrose concentration and pH on the culture medium, and aimed to increase the cellular growth of *Ralstonia solanacearum* RS.

**Table 2:** Matrix of the experimental planning CCRD 2^2^. Coded and real levels as well as values of the response variables obtained for *R*. *solanacearum* RS inoculum incubated at 32°C and 250 rpm for 24 h and YM culture medium with sucrose as the carbon source.

Treat	Independent variables		Dependent variables			
	pH	[Sucrose]	OD_600nm_	DCW (g.L^-1^)	Sugar (g.L^-1^)	P(3HB) (%)
1	-1 (5.8)	-1 (15)	4.7	1.22	4.25	18.53
2	+1 (7.2)	-1 (15)	9.0	2.34	8.37	28.32
3	-1 (5.8)	+1 (45)	20.3	5.27	16.98	23.12
4	+1 (7.2)	+1 (45)	2.0	0.52	2.37	19.71
5	-1.41 (5.7)	0 (30)	20.6	5.35	15.73	45.62
6	+1.41 (7.3)	0 (30)	7.4	1.92	6.52	31.63
7	0 (6.5)	-1.41 (12.9)	11.2	2.91	8.11	37.39
8	0 (6.5)	+1.41 (47.1)	15.5	4.03	12.35	41.39
9	0 (6.5)	0 (30)	17.7	4.54	13.78	41.99
10	0 (6.5)	0 (30)	17	4.52	13.16	39.15
11	0 (6.5)	0 (30)	16.2	4.53	12.87	39.33

Treat, treatments; OD_600nm_, optical density; DCW, dry cell weight; sugar, sugar consumption; P(3HB), poly(3-hydroxybutyrate).

The highest cell growth for sucrose, estimated by OD_600nm_ (20.3 and 20.6) and DCW (5.27 and 5.35) values, was obtained in culture medium treatments 3 and 5, as well as higher sugar consumption, 16.98 and 15.73, respectively. These treatments had pH values below or equal to the minimum value and sucrose concentrations above or equal to the centre point. A significant and predictive mathematical model was generated with 95% confidence, and the determination coefficients (R^2^) were 0.88, 0.89 and 0.93 for the dependent variables OD_600nm_, DCW and sugar consumption, respectively, using sucrose as the carbon source in the culture medium. The coefficient of determination (R^2^) measured the proportion of the total response variation that is explained by the model. Models with an R^2^<0.60 should be used only as trend indicators and never for predictive purposes [31–33].

For the production of P(3HB), the independent variables did not show a significant effect on the evaluated ranges. However, the polymer accumulation was higher under some conditions, particularly in treatment 5 (45.6%), in which the cell growth was also high. However, treatment 3 resulted in high OD_600nm_ (20.3) and DCW (5.27) values and had a lower concentration of P(3HB) (23.12%). This result indicated that such inoculum may result in greater polymer accumulation under suitable conditions. As treatment 3 resulted in OD_600nm_ and DCW values similar to those of treatment 5 but with an approximately 50% lower polymer concentration, it can be assumed that the DCW obtained in treatment 3 has a higher percentage of material due to the cells themselves. Since P(3HB) is an intracellular polymer, an inoculum with a high numerical cell concentration is very desirable. On the other hand, the high polymer concentration obtained following treatment 5 strongly suggests the suitability of using organic YM medium or variants in the production phase of P(3HB). Statistical analysis showed that 95% significant regression coefficients were considered in the mathematical models proposed to represent Eq 2 (OD_600nm_), Eq 3 (DCW) and Eq 4 (sucrose consumption) of the cultures as a function of pH and sucrose concentration. Eq 2, 3 and 4 are as follows:

$$OD600nm\;=\;16.98\;-\;8.18\;\times \;pH\;-\;5.34\;\times \;pH^{2}\;+\;3.68\;\times \;S\;-\;6.00\;\times \;S^{2}\;-\;11.30\;\times \;pH\;\times \;S$$ (2)

$$DCW\;=\;4.53\;-\;2.12\;\times \;pH\;-\;1.51\;\times \;pH^{2}\;+\;0.95\;\times \;S\;-\;1.68\;\times \;S^{2}\;-\;2.93\;\times \;pH\;\times \;S$$ (3)

$$Sucrose\;Consumption\;=\;13.28\;-\;5.89\;\times \;pH\;-\;3.51\;\times \;pH^{2}\;+\;3.19\;\times \;S\;-\;4.41\;\times \;S^{2}\;-\;9.36\;\times \;pH\;\times \;S$$ (4)

**Fig 2:**
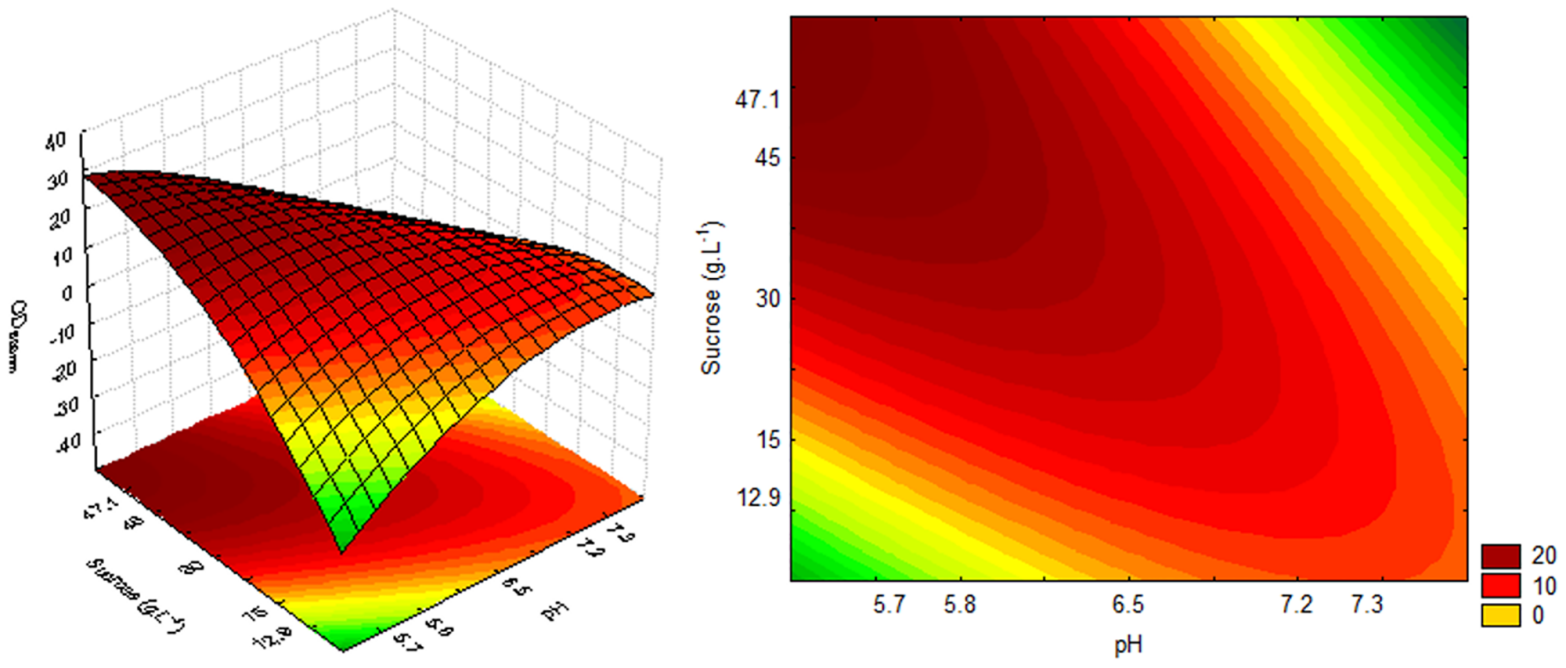
Response and contour surfaces as a function of sucrose concentration and pH for OD_600nm_.

**Fig 3:**
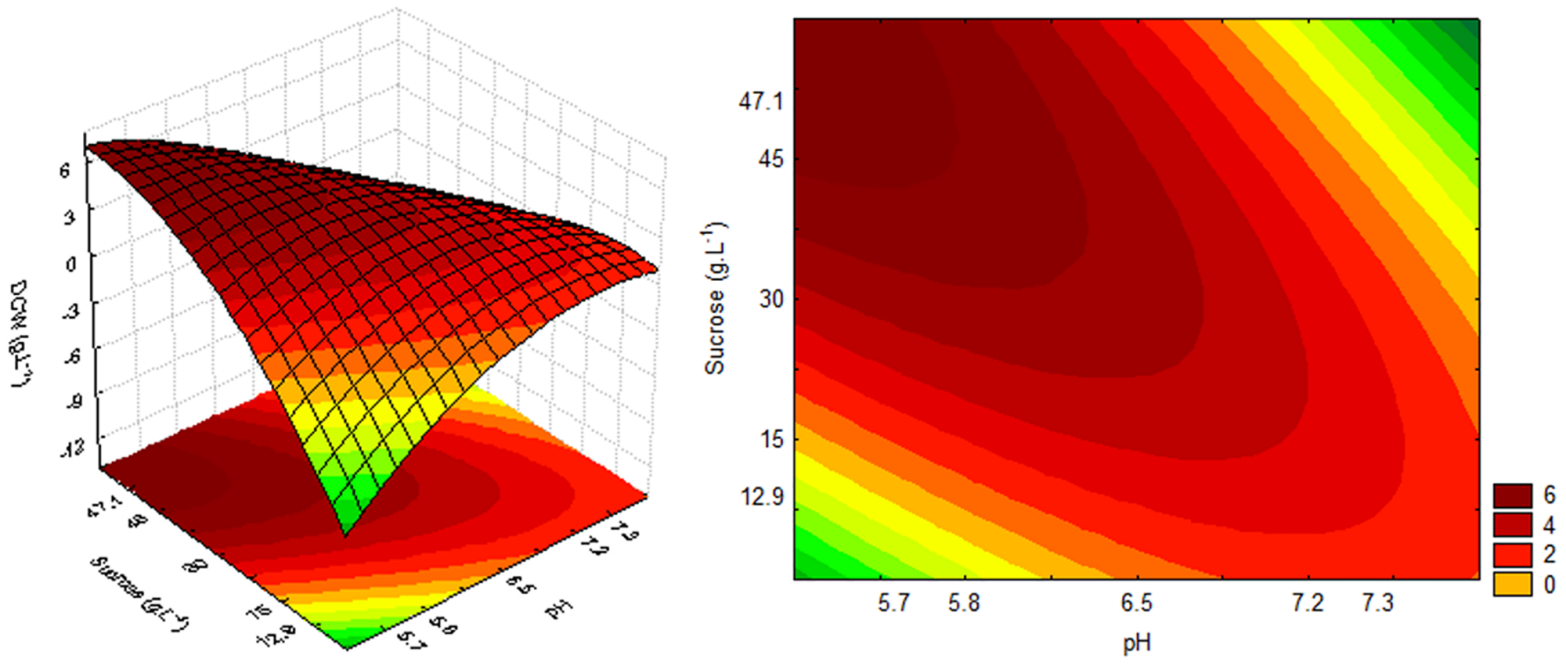
Response and contour surfaces as a function of sucrose concentration and pH for DCW.

**Fig 4:**
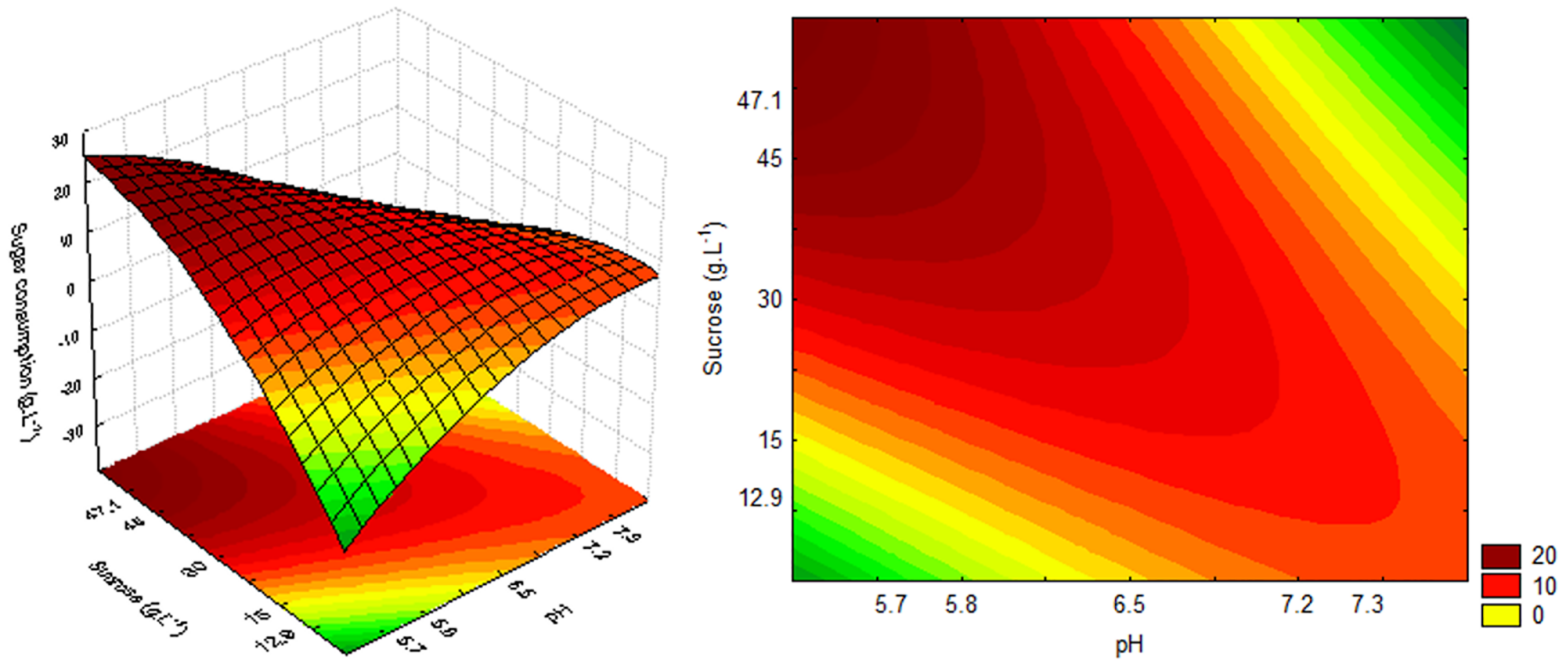
Response and contour surfaces as a function of sucrose concentration and pH for sugar consumption.

The response and contour surfaces for cultures using sucrose, based on the model generated by Eqs 2, 3 and 4, are shown in Figs 2, 3 and 4, respectively.

The response surfaces (Figs 2, 3 and 4) indicate that the highest cell growth in the cultivation using sucrose, OD_600nm_ 20 and DCW 6 g.L^-1^, as well as higher sugar consumption (20 g.L^-1^), can be obtained with a high sugar concentration and an acid pH.

Table 3 presents the results of the experiment, in which glucose was used as the carbon source, generated by the application of the statistical central composite rotational design (CCRD 2^2^).

**Table 3:** Matrix of the experimental planning CCRD 2^2^. Coded and real levels as well as values of the response variables obtained for *R*. *solanacearum* RS inoculum incubated at 32°C and 250 rpm for 24 h and YM culture medium with glucose.

Treat	Independent variables		Dependent variables			
	pH	[Glucose]	OD_600nm_	DCW (g.L^-1^)	Sugar (g.L^-1^)	P(3HB) (%)
1	-1 (5.5)	-1 (10)	12.5	2.74	9.67	37.87
2	+1 (9.5)	-1 (10)	7.7	1.68	7.69	38.99
3	-1 (5.5)	+1 (50)	3.0	0.22	1.78	28.99
4	+1 (9.5)	+1 (50)	1.9	0.40	3.07	23.21
5	-1.41 (5.2)	0 (30)	8.8	1.56	8.95	29.32
6	+1.41 (9.8)	0 (30)	3.0	0.48	2.15	18.99
7	0 (7.5)	-1.41 (7.2)	11.4	2.69	6.95	37.19
8	0 (7.5)	+1.41 (52.8)	1.7	0.78	4.28	11.04
9	0 (7.5)	0 (30)	10.3	2.38	11.06	35.23
10	0 (7.5)	0 (30)	10.6	2.48	10.46	29.10
11	0 (7.5)	0 (30)	10.8	2.53	10.87	32.80

Treat, treatment; OD_600nm_, optical density; DCW, dry cell weight; sugar, sugar consumption; P(3HB), poly(3-hydroxybutyrate).

Table 3 shows that the highest cell growth using glucose, estimated using the OD_600nm_ (12.5) and DCW (2.74) values, was obtained in treatment 1, with culture medium at acidic pH and a sugar concentration at the minimum values evaluated. It was also observed that in this treatment, almost all the available sugar was consumed (9.67 g.L^-1^).

The generated mathematical model was significant, with 95% confidence, and predictive with coefficients of determination (R^2^) 0.99, 0.96 and 0.85 for the dependent variables OD_600nm_, DCW and sugar consumption, respectively, using glucose as a carbon source in the organic culture medium YM. The measured ranges of pH and sugar concentration had no significant effect on the accumulation of P(3HB). Similar to the accumulation with sucrose cultivation, high accumulation of P(3HB) was obtained for glucose, and even higher accumulations were observed in the treatments in which sugar concentrations were at the lowest (T1, T2 e T7).

Statistical analysis revealed that 95% significant regression coefficients were considered in the mathematical models proposed to represent Eq 5 (OD_600nm_), Eq 6 (DCW) and Eq 7 (glucose consumption) of the cultures, as a function of pH and glucose concentration. Eqs 5, 6 and 7 are as follows:

$$OD600nm\;=\;10.57\;-\;3.53\;\times \;pH\;-\;4.66\;\times \;pH^{2}\;-\;7.26\;\times \;G\;-\;4.00\;\times \;G^{2}\;+\;1.85\;\times \;pH\;\times \;G$$ (5)

$$DCW\;=\;2.46\;-\;0.60\;\times \;pH\;-\;1.51\;\times \;pH^{2}\;-\;1.63\;\times \;G\;-\;0.79\;\times \;G^{2}\;+\;0.62\;\times \;pH\;\times \;G$$ (6)

$$Glucose\;consumption\;=\;10.80\;-\;2.58\;\times \;pH\;-\;5.28\;\times \;pH^{2}\;-\;4.08\;\times \;G\;-\;5.21\;\times \;G^{2}\;+\;1.63\;\times \;pH\;\times \;G$$ (7)

**Fig 5:**
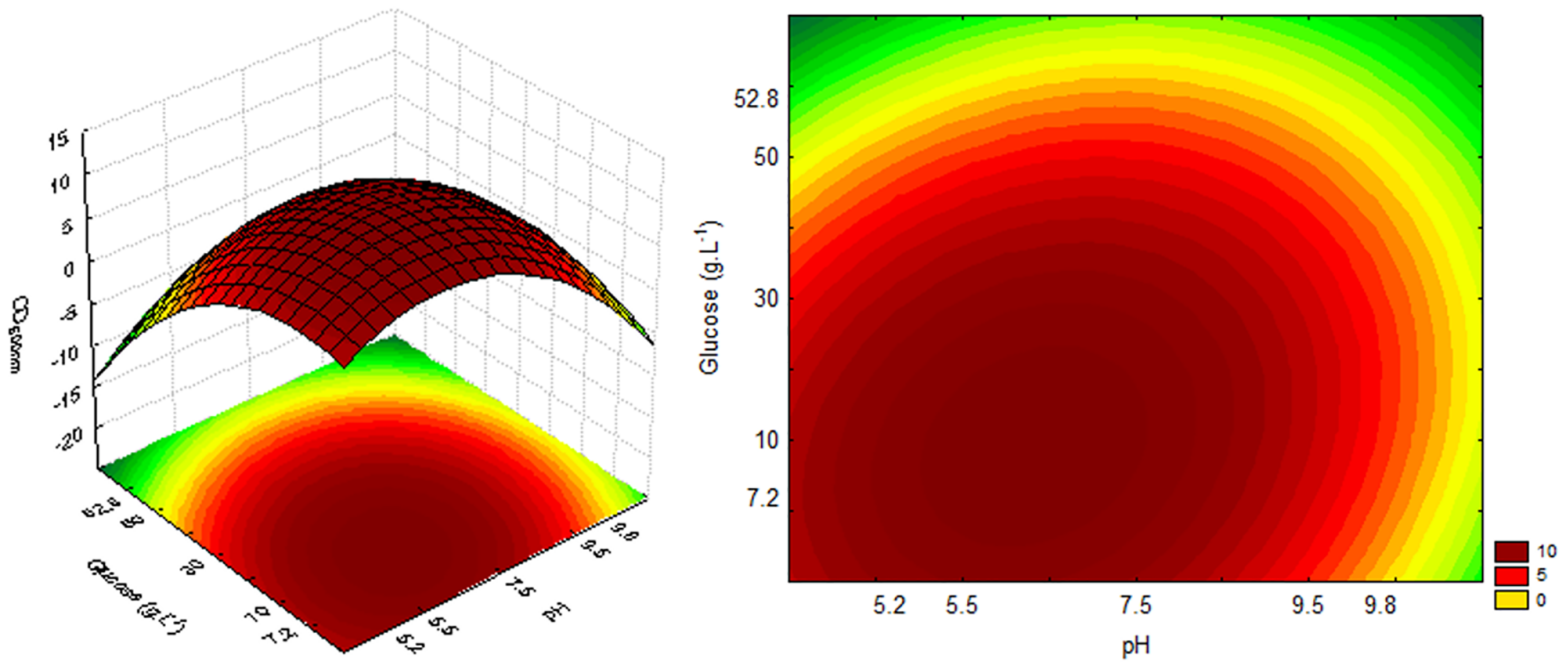
Response and contour surfaces as a function of glucose concentration and pH for OD_600nm_.

**Fig 6:**
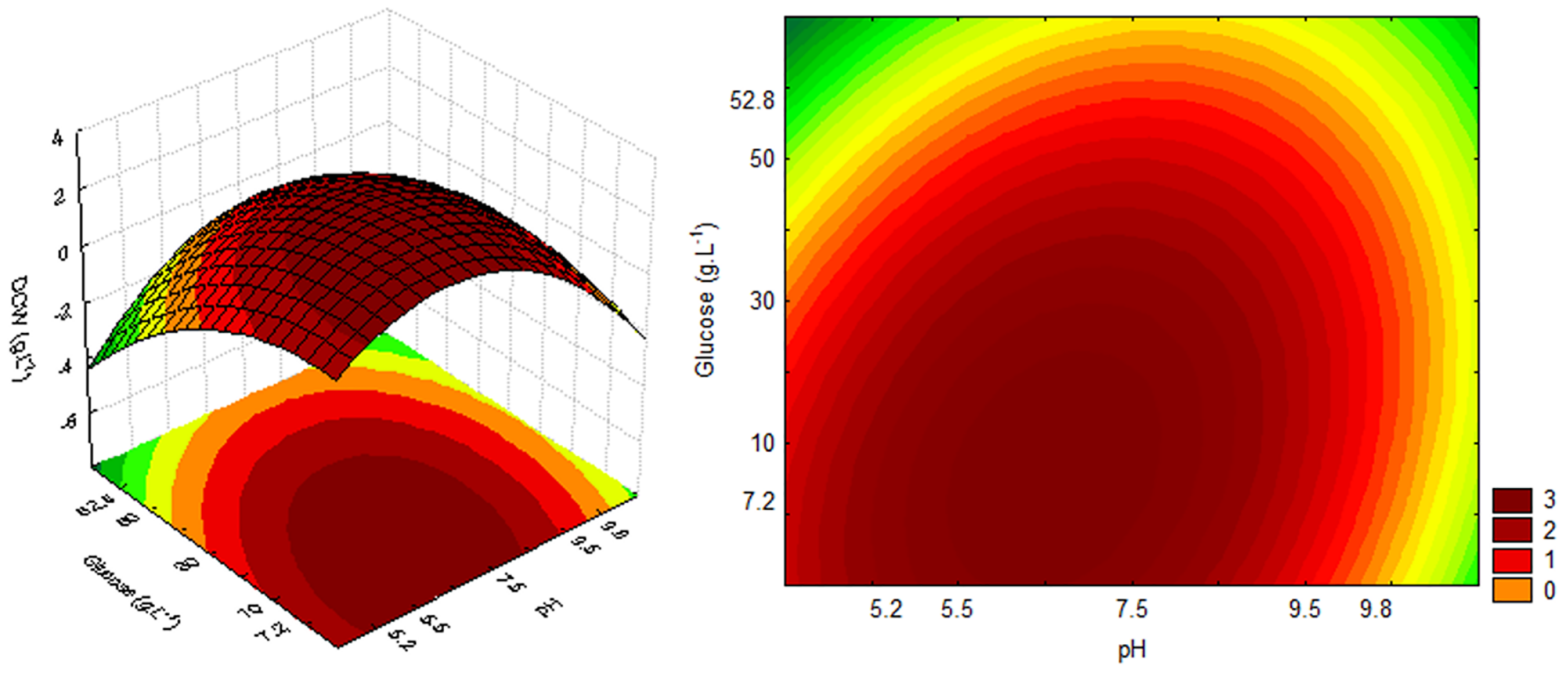
Response and contour surfaces as a function of glucose concentration and pH for DCW.

**Fig 7:**
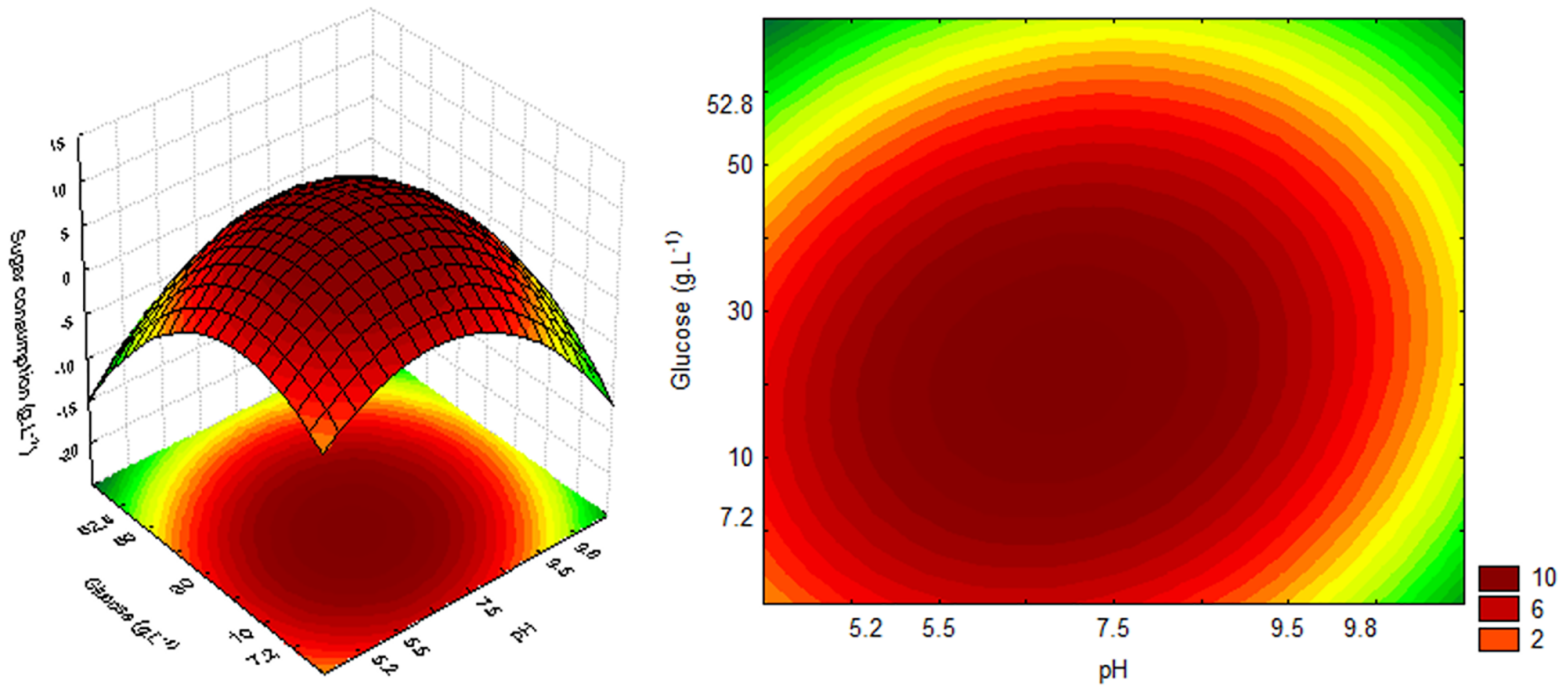
Response and contour surfaces as a function of glucose concentration and pH for sugar consumption.

The response and contour surfaces for the cultures using glucose, which were based on the model generated by Eqs 5, 6 and 7, are shown in Figs 5, 6 and 7 respectively.

From the response surfaces (Figs 5, 6 and 7), it was estimated that the highest cell growth (OD_600nm_ 10 and DCW 3 g.L^-1^), as well as higher sugar consumption (10 g.L^-1^), in the culture using glucose can be obtained with a sugar concentration and a pH between the minimum values and central point. The inhibitory effect on cell growth may also be observed when high glucose concentrations and pH are used.

The use of pH 7 for culture medium in the inoculum phase has been indicated for the genus *Ralstonia* [19,34]. However, for *R*. *solanacearum* RS, the present study found that the appropriate pH depends on the type of sugar and its concentration.

Varying results have been reported regarding the type and concentration of the sugar used for the different P(3HB)-producing microorganisms. Some examples include TFY medium composed of fructose 5 g.L^-1^ for *Ralstonia eutropha* ATCC 17697 [34]; BCM composed of sucrose 10 g.L^-1^ for *Bacillus mycoides* RLJ B-017 [11]; nutrient broth composed of sucrose 10 g.L^-1^ for *Bacillus megaterium* BA-019 [15–16]; nutrient broth composed of glycerol 20 g.L^-1^ for *Bacillus megaterium* [18] and Luria-Bertani (LB) supplemented with 30 g.L^-1^ of glycerol for *Cupriavidus necator* [35]. However, these authors did not report the values of the bacterial concentration in the inoculum phase; as such, it is not possible to compare the outputs of these studies with that of the current study.

The microorganisms used to produce PHAs can be divided into two groups. The first group (I) consists of microorganisms that produce the polymer in conditions of excess carbon. Their growth is interrupted or reduced by the lack of essential nutrients such as N, P, Mg, K, S or O [36]. *Ralstonia* sp. is considered to belong to group I; however, in the current study, it was possible to verify this bacterium’s excessive accumulation of P(3HB) in the inoculum phase, second group (II) [17,36], which may be a specific characteristic of the species investigated in the study.

Factorial design is a common practice in biotechnology and an important tool to determine the optimal process conditions. This technique is advantageous compared to the conventional method, which handles a single parameter per trial; as such, an approach fails to locate optimal conditions due to its failure to consider the effect of possible interactions between factors. Various research workers have applied the Factorial technique, especially for the optimization of culture conditions [37–41] and for the determination of optimal values for processing parameters [42–44].

In the experimental design, the independent variables were substituted by the coded values, obtaining the predicted values and the standard deviations (Tables 4 and 5).

**Table 4:** Responses of the complete experimental design 2^2^ for OD_600nm_, DCW and sugar consumption obtained from the inoculum of *R*. *solanacearum* RS with sucrose, experimentally observed and predicted by the model, and their respective standard deviations.

Treat	pH	Sucrose	OD_600nm_			DCW			Sugar Consumption		
			Obs	Model	SD(%)	Obs	Model	SD(%)	Obs	Model	SD(%)
1	-1	-1	4.7	-1.16	124.7	1.22	-0.42	134.4	4.25	-1.3	130.6
2	+1	-1	9.0	5.08	43.6	2.34	1.2	48.7	8.37	5.64	32.6
3	-1	+1	20.3	28.8	-41.9	5.27	7.34	-39.3	16.98	23.8	-40.16
4	+1	+1	2.0	-10.16	608.0	0.52	-2.76	630.8	2.37	-6.7	382.7
5	-1.14	0	20.6	17.89	13.2	5.35	4.52	15.5	15.73	14.6	7.2
6	+1.14	0	7.4	-5.17	169.9	1.92	-1.46	176.0	6.52	-2.0	130.7
7	0	-1.14	11.2	-0.14	101.3	2.91	-0.15	105.2	8.11	0.01	99.9
8	0	+1.14	15.5	10.24	33.9	4.03	2.53	37.2	12.35	9.01	27.0
9	0	0	17.7	16.98	4.1	4.54	4.53	0.2	13.78	13.28	3.63
10	0	0	17	16.98	0.1	4.52	4.53	-0.2	13.16	13.28	-0.9
11	0	0	16.2	16.98	-4.8	4.53	4.53	0.0	12.87	13.28	-3.18

Treat, treatments; Obs, responses experimentally observed; model, results of predictive model; SD, relative standard deviation; OD_600nm_, optical density; DCW, dry cell weight.

**Table 5:** Responses of the complete experimental design 2^2^ for OD_600nm_, DCW and sugar consumption, obtained from the inoculum of *R*. *solanacearum* RS with glucose, experimentally observed and predicted by the model and their respective standard deviations.

Treat	pH	Sucrose	OD_600nm_			DCW			Sugar Consumption		
			Obs	Model	SD(%)	Obs	Model	SD(%)	Obs	Model	SD(%)
1	-1	-1	12.5	14.55	-16.4	2.74	3.01	-9.9	9.67	8.6	11.1
2	+1	-1	7.7	3.79	50.8	1.68	0.57	66.1	7.69	0.18	97.6
3	-1	+1	3.0	-3.67	222.3	0.22	-1.49	777.3	1.78	-2.82	258.4
4	+1	+1	1.9	-7.03	470.0	0.40	-1.45	462.5	3.07	-4.72	253.7
5	-1.14	0	8.8	6.29	28.5	1.56	0.31	80.1	8.95	3.94	56.0
6	+1.14	0	3.0	-3.67	222.3	0.48	-1.39	389.6	2.15	-3.34	255.3
7	0	-1.14	11.4	12.86	-12.8	2.69	3.19	-18.6	6.95	6.2	10.8
8	0	+1.14	1.7	-7.62	548.2	0.78	-1.41	280.8	4.28	-5.3	223.8
9	0	0	10.3	10.57	-2.6	2.38	2.46	-3.4	11.06	10.8	2.3
10	0	0	10.6	10.57	0.3	2.48	2.46	0.8	10.46	10.8	-3.2
11	0	0	10.8	10.57	2.1	2.53	2.46	2.8	10.87	10.8	0.6

Treat, treatments; Obs, responses experimentally observed; model, results of predictive model; SD, relative standard deviation; OD_600nm_, optical density; DCW, dry cell weight.

By analysing the results of the predicted model equations and the experimental results for the OD_600nm_, DCW and sugar consumption values, responses were found at very similar central points with a low standard deviation. This finding indicates that the model is valid for both carbon sources that were analysed. It was below the deviation in treatment 5, which used sucrose and for which a higher concentration of biomass was obtained with the combination of the negative axial point for the pH and the centre point for the sugar concentration. Treatment 1 predicts a high biomass (using glucose) by combining the minimum values of the independent variables.

Thus, accumulation of the inoculum depended on optimizing the different combinations of pH and sugar concentrations. This can be achieved by setting the independent variables at one or more of these centre points for both carbon sources and a combination of additional values mentioned for each type of sugar in the optimization range.

## Conclusions

Higher cell growth and the consequent higher yield of P(3HB) were obtained using sucrose (45 to 47.1 g.L^-1^) as a carbon source in an acidic pH (5.7 to 5.8) medium. The ideal pH depends on the concentration and type of sugar used. Due to the significant accumulation of polymer while the cells were still in the cell growth phase, the accumulating microorganism P(3HB) *Ralstonia solanacearum* RS can be classified as having type II metabolism in relation to the accumulation phase of the polymer.
